# Deep Brain Stimulation Outcomes in Parkinson's Disease Patients with Cognitive Impairment: Implications and Considerations

**DOI:** 10.1002/mdc3.13819

**Published:** 2023-06-30

**Authors:** Alberto Romagnolo, Margherita Fabbri, Carlo Alberto Artusi, Maurizio Zibetti, Leonardo Lopiano, Elisa Montanaro

**Affiliations:** ^1^ Department of Neuroscience “Rita Levi Montalcini” University of Turin Turin Italy; ^2^ SC Neurologia 2U, AOU Città della Salute e della Scienza Turin Italy; ^3^ Department of Neurosciences, Clinical investigation Center CIC 1436, Parkinson Toulouse Expert Center, NS‐Park/FCRIN Network and NeuroToul COEN Center Toulouse University Hospital, INSERM, University of Toulouse 3 Toulouse France

**Keywords:** Parkinson's disease, deep brain stimulation, cognitive impairment, dementia

Deep brain stimulation (DBS) has profoundly changed the treatment of Parkinson's Disease (PD). Although the selection of patients for DBS is currently driven by validated criteria,^1^ the patient's cognitive status—in particular the presence of mild cognitive impairment (MCI)—still generates discussion among clinicians. In their recent work, Block and colleagues^2^ compared the DBS outcomes between patients with “moderate” cognitive impairment (CI) (a sort of intermediate passage between MCI and overt dementia) and patients with normal cognition (NC). The authors found good clinical outcomes in CI patients after 12 months of follow‐up, comparable to those of NC ones, and postulated that their results could expand the current cognitive cut‐offs for DBS selection. We read with interest this well‐designed paper, and we firmly argue that any new insight on DBS outcome in patients with CI will help in informing clinicians about the best way to select patients for surgery. However, as correctly stated by the authors, this study is burdened by incontrovertible limitations, that must be considered before transposing its findings into current clinical practice. First, grading the efficacy of DBS in CI patients solely on motor and fluctuations outcomes is partially limiting, since they are widely demonstrated effects of DBS, not strictly related to the cognitive status. Second, though not reaching the statistical threshold, CI patients presented with higher incidence of potentially disabling side effects (ie, 2‐fold, 3‐fold, 1.4‐fold, and 4‐fold higher incidence of intracranial lesions, lead migration/misplacement/malfunction, neurological and psychiatric side effects, respectively). Third, the definition of “moderate” CI which, although well described in the Methods section, does not correspond to a precise clinical diagnosis, covering a broad spectrum between MCI and dementia.^3^ Fourth, and most critical considering the study target, the issue related to the cognitive outcome: we believe that its evaluation by means of UPDRS‐I, with approximately 50% of missing data, after only 12 months from surgery, cannot comprehensively describe the real evolution of the patient's cognitive status. In a previous study comparing the long‐term DBS outcomes in MCI vs. NC patients, we also found good motor outcomes and unchanged cognitive status—measured with a comprehensive neuropsychological battery—after one year in both groups, but a longer follow‐up disclosed a significantly faster conversion to dementia among MCI versus NC patients (6 vs. 11 years after DBS).^4^

After more than two decades of DBS in PD, the correct identification of the most suitable patients for surgery is still a matter of debate. Any new information aimed at filling the knowledge gap on which PD features are most critical in predicting DBS outcome is of utmost importance,^5^ and the work from Block and colleagues certainly follows this path. However, caution is needed in interpreting data about cognitively impaired patients, due to the risk of focusing our judgment mainly on motor outcome, forgetting the high rate of surgical side effects, suboptimal functional outcomes, and mid‐to‐long‐term risk of cognitive worsening. Long‐term studies with comprehensive neuropsychological and quality of life evaluations are needed to inform clinical practice standards.

## Author Roles

(1) Research project: A. Conception, B. Organization, C. Execution; (2) Statistical Analysis: A. Design, B. Execution, C. Review and Critique; (3) Manuscript Preparation: A. Writing of the first draft, B. Review and Critique.

A.R.: 1A, 1B, 1C, 3A.

M.F.: 1A, 3B.

C.A.A.: 1A, 3B.

M.Z.: 1A, 3B.

L.L.: 1A, 3B.

E.M.: 1A, 1B, 1C, 3B.

## Disclosures

**Ethical Compliance Statement:** The authors confirm that the approval of an institutional review board was not required for this work. The authors confirm that informed patient consent was not required for this work. We confirm that we have read the Journal's position on issues involved in ethical publication and affirm that this work is consistent with those guidelines.

**Founding Sources and Conflict of Interest:** No specific funding was received for this work. The authors declare that there are no conflicts of interest relevant to this work.

**Financial Disclosures for the Previous 12 Months:** A.R. speaking honoraria from Bial and Zambon; travel grants from Chiesi Farmaceutici. M.F. speaking honoraria from AbbVie, Bial, and Orkyn; consultancies for Bial and LVL médical. C.A.A. speaking honoraria from Bial, Zambon, and Lusofarmaco. M.Z. speaking honoraria from AbbVie, Bial, and Medtronic. L.L. speaking honoraria from AbbVie and Bial. E.M. no disclosures to report.
